# Utility of the Predictive Summary Index for Comparison of Diagnostic Protocols in Myasthenia Gravis With Repetitive Nerve Stimulation and Concentric Needle Jitter

**DOI:** 10.1002/brb3.71016

**Published:** 2025-10-29

**Authors:** Peter Trillenberg, Daphne Schepers‐von Ohlen

**Affiliations:** ^1^ Department of Neurology University Hospital of Schleswig‐Holstein, Campus Lübeck Lübeck Germany; ^2^ Department of Radiation Oncology University Hospital of Schleswig‐Holstein, Campus Lübeck Lübeck Germany

**Keywords:** concentric needle jitter, electrophysiology, myasthenia, repetitive nerve stimulation, single fiber electromyography

## Abstract

**Introduction:**

As sensitivity of a test can be increased at the expense of specificity, a quality measure taking into account both parameters is desirable. The “predictive summary index” (PSI) is calculated from the prevalence of the disease, sensitivity and specificity. We characterize diagnostic yield of repetitive nerve stimulation (RNS) and stimulated concentric needle jitter (CNJ) in the diagnosis of myasthenia gravis (MG) with PSI. From PSI, the number of tests that is required for the prediction of one correct diagnosis (“number needed to predict” [NNP] in analogy to the number needed to treat) can be calculated as 1/PSI.

**Methods:**

Among patients consecutively referred to the electrophysiology laboratory for a workup of a disorder of the neuromuscular junction (NMJ), we identified 35 patients with MG (clinical diagnosis supported by antibody findings or treatment response) and 85 patients with diagnosis unrelated to the NMJ in whom both RNS and stimulated CNJ had been performed.

**Results:**

The PSI was 0.82 and 0.53 for CNJ and RNS, respectively. The NNPs were 1.22 for CNJ and 1.89 for RNS. This means that 1.49 patients have to be tested with CNJ instead of RNS to arrive at one correct diagnosis. In data from the literature, PSI illustrates that 7% as a cut‐off in RNS can be helpful and that amplitude decrement is more reliable than area decrement.

**Conclusion:**

CNJ was more reliable in the diagnosis of MG than RNS. PSI provides a rational quantitative framework to discuss both the validity of different techniques and their cost in terms of time spent to arrive at a correct diagnosis.

## Graphical abstract

We use retrospective data from our electrophysiology laboratory to show that to diagnose or rule out myasthenia gravis one in every 1.22 concentric needle jitter tests and one in 1.89 repetitive nerve stimulations tests leads to the correct prediction.

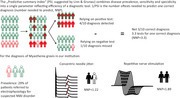

## 1 Introduction

The diagnosis of disorders of the neuromuscular junction (NMJ) and of myasthenia gravis (MG) in particular relies on clinical assessment, including treatment response, antibody (AB) testing, and electrophysiology. The correct clinical characterization seems to have the largest impact on a correct diagnosis (Andrapalliyal et al. 2023).

For the electrophysiological confirmation of a disorder of the NMJ, two techniques are available (American Association of Electrodiagnostic Medicine Quality Assurance Committee 2001; Sanders et al. 2019): Repetitive nerve stimulation (RNS) detects failure of some NMJs after repeated stimulation by a decrease of the amplitude of the compound muscle action potential (CMAP) after its nerve stimulation (decrement). Single fiber (sf) electromyography (sfEMG) rather detects *impeding* failure of NMJs by an increase in variability of the interpotential intervals (IPI) between two muscle fibers of the same motor unit (voluntary sfEMG) or an external (percutaneous nerve stimulation) or internal (intramuscular microaxonal stimulation) electrical stimulus to the nerve controlling a muscle and the action potential of a muscle fiber (stimulated sfEMG) (American Association of Electrodiagnostic Medicine Quality Assurance Committee 2001; Sanders et al. 2019). The sfEMG can be recorded with a specialized sf needle electrode or with a standard concentric EMG needle with small inner core (Ertas et al. 2000) (after increasing the low‐pass filter of the amplifier). The latter avoids reusable needles and drastically reduces costs. However, it is not guaranteed that this setup single muscle fiber action potentials are recorded exclusively (as opposed to compound extracellular correlates of action potentials of a few muscle fibers) because the area of recording is larger with the concentric needle. Therefore, this technique is referred to as “concentric needle jitter” (CNJ).

RNS is easy to perform but can be remarkably low in sensitivity at least in ocular myasthenia (Zambelis et al. 2011). sfEMG is more time‐consuming and technically more challenging. Very high sensitivities (99% in generalized and 97% in ocular myasthenia) have been reported for recording with the specialized sf electrode (Sanders and Stalberg 1996).

**FIGURE 1:**
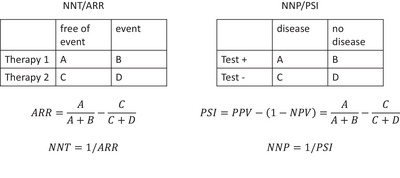
Analogy between number needed to treat (NNT) as calculated from the absolute risk reduction (ARR) on one hand, and the number needed to predict (NNP) as calculated from the predictive summary index (PSI) on the other hand.

The diagnostic impact of either test depends on being reliably positive in patients with MG (described by sensitivity) and reliably negative in the absence of a disorder of the NMJ (high specificity). The “predictive summary index” (PSI) elegantly weights sensitivity and specificity with the prevalence of the diagnosis in the sample into a single number reflecting the average gain in information by performing a test (Linn and Grunau 2006). The PSI is calculated as the difference between the positive predictive value (PPV) and the false negative rate. The latter equals 1‐negative predictive value (1‐NPV). Therefore, PSI = PPV − (1‐NPV) = PPV + 1‐NPV. The PSI can be interpreted in analogy to the number needed to treat (NNT) (Cook and Sackett 1995) as follows: The NNT is calculated as the inverse of the “absolute risk reduction” (ARR). ARR quantifies the increase of probability for a favorable outcome with a new therapy as compared to a standard therapy. In analogy, PSI reflects the increase of disease probability with a positive test as compared to a negative test. In a next step, the NNT is calculated as 1/ARR, indicating “the number of patients that need to be treated with the new therapy as opposed to the standard therapy to achieve one additional favorable outcome.” In analogy, the “number needed to predict” (NNP) is calculated as 1/PSI and represents “the number of patients that need to be tested positive as opposed to be tested negative to end up with one additional correct diagnosis of the disease.” Thus, to see the analogy of NNT and PSI, the two therapies are replaced by positive and negative test result, respectively, and the freedom from event is replaced by diagnosis of the disease (Figure 1) (Linn and Grunau 2006).

We sought to compare PSI for CNJ and RNS as performed in our institution and as calculated from the literature, show its variation with the prevalence of MG in our sample and in samples from the literature, and discuss whether recording in additional muscles or changes in the cut‐off to abnormal help to improve PSI as a measure of overall diagnostic performance.

## 2 Methods

From September 2015 to March 2020, we retrospectively identified consecutive patients in whom both RNS and CNJ had been carried out at the electrophysiology laboratory of our institution. We excluded three patients with LEMS and one patient with botulinum toxin as causes of weakness. The remaining 128 patients were assigned either to the MG group or to the control group. To assign a patient to the myasthenia group, we required (1) fluctuating weakness, including isolated weakness of the extraocular muscles or ptosis, and (2) documented treatment response to acetylcholine esterase inhibitors, or (3) detection of antibodies (ACh receptor, MuSK, or LRP4). These criteria could be verified in the charts of 35 patients. In 89 cases, the clinicians diagnosed another disorder not related to the NMJ. In four of these patients, ABs against the acetylcholine receptor in low concentration or antibodies against Ca channels could be demonstrated. These patients were excluded from the control group. The assignment of patients to MG and the control group is illustrated in Figure S1.

For RNS, usually the nasalis muscle and the trapezius muscle of one side were tested. Decrement was calculated from the peak‐to‐peak amplitude of the fifth as compared to the first CMAP (American Association of Electrodiagnostic Medicine Quality Assurance Committee 2001). The cutoff for an abnormal result was set at 7% decrement (Lamb and Rubin 2020) which has been shown to offer increased sensitivity at identical specificity as compared to the otherwise popular choice of 10% (American Association of Electrodiagnostic Medicine Quality Assurance Committee 2001). If the decrement was abnormal in one muscle, RNS was considered positive.

The CNJ was recorded from the frontalis muscle with a concentric needle electrode (D0390302511, Spes Medica, Genova, Italy) with recording area 0.02 mm^2^. Muscle contraction was elicited by electrical stimulation of the forehead branch of the facial nerve with surface electrodes at a frequency of 10 Hz (Ertas et al. 1998) with pulses of 0.04 ms width and currents of 10–15 mA. Stimulated sfEMG as opposed to sfEMG with voluntary activation has the advantage that the patient does not have to maintain constant voluntary activation and that the influence of the velocity recovery function is eliminated (Sanders et al. 2019). With the stimulated technique, care has to be taken to identify undesired summation of sf action potentials. Others have concluded that summation can be avoided with appropriate stimulation current in facial muscles (as opposed to large limb muscles) (Kouyoumdjian et al. 2011). Moreover, care has to be taken to avoid increased jitter due to submaximal stimulation of axons.

The signal was amplified in the frequency range 1–10 kHz. This filter setting is standard in CNJ recording to reduce summation of signals from distant muscle fibers (Sanders et al. 2019).

A mean jitter calculated from 30 potentials exceeding 21 µs was considered abnormal (Table 2 in Stalberg et al. 2016). Recordings were made with a Keypoint G4 workstation with Keypoint.Net software (Natus Medical Incorporated, Middleton, Wisconsin, United States).

Sensitivity, specificity, PPV, and NPV were calculated according to standard definitions. The PSI was calculated as PSI = PPV − (1‐NPV). Confidence intervals were calculated with the bootstrap method (Dikta and Scheer 2021). Calculations were carried out with the Statistics and Machine Learning Toolbox of Matlab (MATLAB and Statistics Toolbox Release 2021a, The MathWorks Inc., Natick, Massachusetts, United States) in which a function for the calculation of confidence intervals with the bootstrap method is included (function “bootci” with 10,000 samples and option “Basic percentile method”). For the simple proportions (sensitivity, specificity, PPV, NPV), the CI was crosschecked with the Wilson Score method with continuity correction (Newcombe 1998) (with excellent agreement between both methods to calculate the confidence intervals). For the PSI, we crosschecked our results for the confidence intervals with a method suggested for the Youden index (Shan 2015), again with good agreement (results see Supporting Information section). Note that the PSI is obtained from the Youden index by exchanging disease status for test result in the contingency table underlying the calculation of the statistical indices. These calculations were also carried out in MATLAB. For the difference PSI(CNJ)–PSI(RNS), we exclusively had to rely on bootstrap for two reasons: First, there seems to be a debate how correlations between the two techniques should be handled (Chen et al. 2015, 2016; Reiser and Nakas 2016). Second, the adaptation of a method for the Youden index for the PSI is not straightforward if two techniques are involved.

The study was approved by the ethics committee of the University of Lübeck (reference 20‐202) and has been performed in accordance with the ethical standards of the 1964 Declaration of Helsinki and its later amendments. As we used anonymized data, individual written informed consent for a retrospective analysis was not needed.

## 3 Results

The prevalence of MG among patients referred for a workup of neuromuscular disorders was 29%. Of the 35 MG patients, 23 were male (66%). Age ranged from 21.8 to 88.7 years, with an average of 66.3 years (interquartile range 53.6–78.8 years). Thirteen (37%) of the MG patients had ocular myasthenia. This proportion and age and gender characteristics are similar to data in the literature (Reyes‐Leiva et al. 2025; Wartmann et al. 2023). Antibodies against the ACh receptor were detected in 29/35 (83%) of patients (CNJ positive in 23 of 29, RNS positive in 17 of 29), and MuSK in 2/35 (6%, CNJ and RNS positive in 1 of 2). Three of the MG patients (9%) were negative for AChR and MuSK AB, and two of these were also negative for LRP4 ABs. These three patients were negative on CNJ testing, and two of them were negative on RNS. For one MG patient, AB results were not available (referral from outside).

In the control group, 46% were female, and average age was 61.4 years. In this group (*n* = 85), the most frequent diagnoses were cranial mononeuropathies (*n* = 15), ptosis (*n* = 15), double vision (*n* = 13), and stroke/TIA (*n* = 10). Interestingly, in one of the control patients, Miller–Fisher's syndrome was diagnosed which is known to sometimes impair neuromuscular transmission (Menon et al. 2012). In our case, however, CNJ and RNS were normal.

Our results for statistical indices are summarized in Table 1, and the raw figures are given in three tables in the Supporting Information section. Sensitivity and specificity of CNJ were higher than for RNS. However, this did not reach significance. Only when both were combined (either directly in the Youden index or weighted with prevalence in the PSI), the difference was significant (PSI: 0.82 [CNJ] vs. 0.53 [RNS], 95% CI for difference 0.037–0.441).

**TABLE 1:** Sensitivity, specificity, positive predictive value (PPV), negative predictive value (NPV), and predictive summary index (PSI) (*p*) for repetitive nerve stimulation (RNS) and concentric needle jitter (CNJ) with the 95% confidence intervals (*p*_min_, *p*_max_).

		CNJ			RNS			Difference CNJ‐RNS		
		*p*	*p*_min_	*p*_max_	*p*	*p*_min_	*p*_max_	*p*	*p*_min_	*p*_max_
Sens	bootstrap	0.714	0.559	0.861	0.571	0.400	0.737	0.143	0	0.289
Spec	bootstrap	0.976	0.944	1	0.894	0.864	0.970	0.082	−0.01	0.122
PPV	bootstrap	0.926	0.811	1	0.690	0.565	0.897	0.236	0.006	0.377
NPV	bootstrap	0.892	0.836	0.953	0.835	0.774	0.917	0.057	0.006	0.099
Youden	bootstrap	0.691	0.530	0.840	0.466	0.315	0.670	0.225	0.044	0.358
PSI	bootstrap	0.818	0.696	0.929	0.525	0.400	0.763	0.294	0.037	0.441

## 4 Discussion

The prevalence of MG is low in our sample (29%), as compared to some other studies (Abraham et al. 2017: 40%; Lamb and Rubin 2020: 56%; Giannoccaro et al. 2020: 71%). However, in a sample of patients with suspected ocular myasthenia, the prevalence was even lower (23% [Mercelis and Merckaert 2011]), and in one other sample, the prevalence was similar to ours with 31% (Morren et al. 2016). The prevalence is a direct consequence of the ease of the institution to perform resource‐consuming electrodiagnostic tests for an NMJ disorder even if the suspicion for that kind of cause for a symptom is low.

Next, we briefly compare our estimates of specificity and sensitivity in particular for CNJ with data from the literature. In mixed samples of ocular and generalized MG sensitivities for CNJ range from 67% (voluntary activation, frontalis muscle [Benatar et al. 2006]), 85% (Kouyoumdjian et al. 2011) (stimulated activation, frontalis muscle) to 96% (Sarrigiannis et al. 2006) (orbicularis oculi muscle, voluntary activation; for a more detailed compilation, refer to Table 2 in Sanders et al. 2019). Thus, our CNJ sensitivity is at the lower end but within the spectrum of published data. Also note that a formal test of our sensitivity against the sensitivity in Kouyoumdjian et al. (2011) (who used identical technique and muscle) is negative (chi‐square with Yates‐correction = 1.2, *p* = 0.27). Estimates of specificity are more sparse and range from 96% (Benatar et al. 2006) to 100% (Sarrigiannis et al. 2006) for CNJ.

NPV, PPV, and PSI (as opposed to sensitivity, specificity, and the Youden index) are influenced by the prevalence of MG patients among all patients referred. The advantage of the former indices is that they offer a more precise interpretation of a test result at the site where the data were recorded. On the other hand, this interpretation might not be adequate in another institution with another prevalence of MG even if the sensitivity and specificity of the test do not differ between sites.

The determination of these indices (that are specific for a given disorder) requires the correct classification of the subjects tested as either affected by the given disorder or affected by another disorder. As there is no definitive test for myasthenia, this classification remains less than perfect. We relied on a fixed combination of clinical and laboratory findings. However, the treatment response that is part of this combination is influenced by patient cooperation which can be influenced by placebo effect, as one example of the imperfection of the group definition.

### 4.1 Interpretation of the PSI

On the basis of our estimates for PPV and NPV as summarized in Table 1, consider 1222 fictitious positive tests with CNJ. They predict on average 1222 × PPV = 1132 correctly identified MG cases. If, on the other hand, the same 1222 tests had had negative results, there would still be 1222 × (1‐NPV) = 132 MG cases. These cases would be missed in a protocol that diagnoses MG based on CNJ results. Thus, 1222 positive tests with CNJ predicted an excess of 1000 MG cases, or, equivalently, 1.22 positive CNJ tests correctly predict one case of MG (NNP). The same figure was 1.89 for RNS. The difference of 0.67 of these figures implies that 1/0.67 = 1.49 CNJ tests as opposed to RNS studies need to be performed for one additional correct diagnosis. As also PSI = NPV − (1‐PPV), PSI also reflects the excess probability of the absence of the disease after a negative test result versus the probability of the absence of the disease when the test is positive. Therefore, 1/PSI also represents the number of subjects that have to be tested negative to correctly predict the absence of the disease in one subject. Therefore, NNP can be applied to positive and negative tests.

Cost in terms of time required for testing can be incorporated as follows: Assume that CNJ in one muscle requires 40 min and that RNS in two muscles requires 20 min. Then, one correct prediction requires 40 min × 1.22 = 49 min with CNJ and 38 min with RNS.

### 4.2 Influence of Disease Prevalence

PSI combines NPV and PPV into one index that takes into account both positive and negative test results weighted with the prevalence of the disease.

**FIGURE 2:**
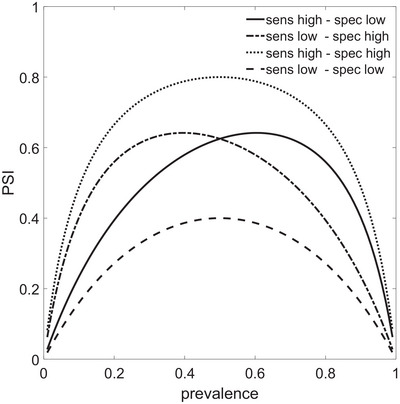
Predictive summary index (PSI) as a function of disease prevalence for high (0.9) or low (0.7) values of sensitivity (Sens) and specificity (Spec) in all possible combinations.

**FIGURE 3:**
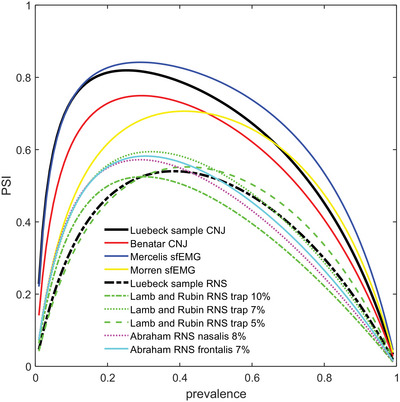
Predictive summary index (PSI) as a function of disease prevalence in various studies of CNJ, sfEMG, and RNS. CNJ, concentric needle jitter; RNS, repetitive nerve stimulation; sfEMG, single fiber electromyography.

PSI is an inverted U‐shaped function of disease prevalence. If specificity exceeds sensitivity, the maximum of the inverted U is shifted to lower prevalences (Figure 2) and vice versa. To illustrate the influence of prevalence, we recalculated PSI with sensitivity and specificity of our study and some related reports (Figure 3). Expressions for NPV and PPV as a function of prevalence, sensitivity, and specificity can be found elsewhere (Wang et al. 2021).

The curves show that with one exception with a very high prevalence (Giannoccaro et al. 2020), the institutions operate close to the maximum of the inverted‐U‐shaped curve that characterizes PSI. For that study, the peak of PSI for sfEMG is shifted to prevalence of 0.5 due to similar sensitivity and specificity. However, for an optimal diagnostic yield, a higher sensitivity even at the expense of specificity would have been necessary. For all other studies, specificity higher than sensitivity and low prevalence fit well with each other. Interestingly, there is one study with a very low specificity of 22% for sfEMG of the orbicularis oculi muscle (Lo et al. 2017) and an excellent sensitivity of 97.3%, and this again is a good choice if the prevalence is as high as in that study (65%).

### 4.3 Is More (Sensitivity) Really More (Information)?

Improvement of sensitivity may be achieved by decreasing cut‐off values or increasing the number of muscles tested. However, this will inevitably compromise specificity. The net benefit of any measure to increase sensitivity can be assessed with the PSI.

If, for example, we had recorded RNS in contralateral muscles as well (thus, a total of four muscles), the sensitivity would increase from Sens to Sens′ = Sens(2‐Sens), and the specificity would decline according to Spec′ = Spec^2^. With our prevalence and appropriate expressions for NPV and PPV, the new PSI increases marginally from 0.52 to 0.54 for RNS. This corresponds to 1/(0.54–0.52 = 50 patients tested for one additional correct diagnosis. For CNJ, the PSI would increase from 0.81 to 0.85 (25 patients required for one more correct diagnosis). Thus, our choices for the routine workup of suspected NMJ disease seem to be a reasonable compromise between effort and result. This conclusion is valid only if the added muscles are similar to the ones already included in sensitivity and specificity. Therefore, adding a clinically more affected muscle might produce more favorable results.

In another study, 77% sensitivity could be achieved by testing the trapezius and anconeus muscles with RNS (Bou Ali et al. 2017). The highest sensitivity of 82% could be achieved by testing at least three muscles (orbicularis oculi, trapezius, and anconeus). Interestingly, the specificity problem caused by inflating the number of muscles tested was controlled by using a decrement cutoff of 10%, which increased the specificity to 100% for every muscle tested. However, due to the low number of controls (*n* = 23) in that study, the 95% confidence interval for the specificity was wide (0.87–1 with the “rule of three” [Hanley and Lippman‐Hand 1983]). Assuming a still high specificity of 95%, testing three muscles on both sides reduces specificity to 78%. The deltoid muscle has been reported to have a higher decrement than M. nasalis, M. anconeus, M. trapezius, or M. abductor dig. V, and thus, with identical cut‐off, a higher sensitivity (Amandusson et al. 2017). However, specificity was not analyzed in that study, and thus, the net effect of the inclusion of this muscle on PSI cannot be calculated.

For RNS, the influence of the cut‐off for the decrement on sensitivity and specificity has been addressed (Lamb and Rubin 2020). The authors conclude that lowering the threshold from 10% to 7% increases sensitivity without a loss in specificity, but that a further decrease compromises specificity. With PSI, this can be substantiated as follows: In that dataset, the PSIs for the amplitude decrement in the trapezius muscle with cut‐offs of 5%, 7%, and 10%, respectively, are 0.52, 0.51, and 0.42, respectively. However, in another study also concluding that a cut‐off of 7%–8% is to be preferred (Abraham et al. 2017), PSI increases as 0.29, 0.57, 0.64, and 0.77 with decrement cut‐offs of 7%, 8%, 9%, and 10%, respectively, in the nasalis muscle. The authors’ conclusion was probably based on the Youden index that peaked at 8% cut‐off. Switching from amplitude to area decrement (with a dramatic increase in sensitivity) (Lamb and Rubin 2020) is not helpful: For the trapezius muscle and a cut‐off of 7%, the PSI is 0.52 (amplitude) versus 0.40 (area).

## 5 Conclusion

In summary, the PSI provides a quantitative tool to discuss various comparisons of diagnostic performance in the field of disorders of the NMJ. It illustrates the diagnostic yield of CNJ and helps to balance it against the increased expenditure of time.

## Author Contributions

**Peter Trillenberg**: conceptualization, writing – original draft, software, formal analysis, methodology, validation. **Daphne Schepers‐von Ohlen**: writing – review and editing, methodology, investigation.

## Funding

The authors have nothing to report.

## Conflicts of Interest

The authors declare no conflicts of interest.

## Peer Review

The peer review history for this article is available at https://publons.com/publon/10.1002/brb3.71016.

## Supporting information




**Supplementary Material**: brb371016‐sup‐0001‐TableS1‐S4.docx

## Data Availability

The data that support the findings of this study are available from the corresponding author upon reasonable request.
