# Supplementary material for: Utility of the Predictive Summary Index for Comparison of Diagnostic Protocols in Myasthenia Gravis With Repetitive Nerve Stimulation and Concentric Needle Jitter
Source: Brain Behav. 2025 Oct 29;15(11):e71016. doi: 10.1002/brb3.71016 (PMC12571967; doi:10.1002/brb3.71016)
Supplement: Supplementary file 1 — Supplementary Material: brb371016‐sup‐0001‐TableS1‐S4.docx [file BRB3-15-e71016-s001.docx]

**Supplementary Material**

**Supplementary Figure 1:**


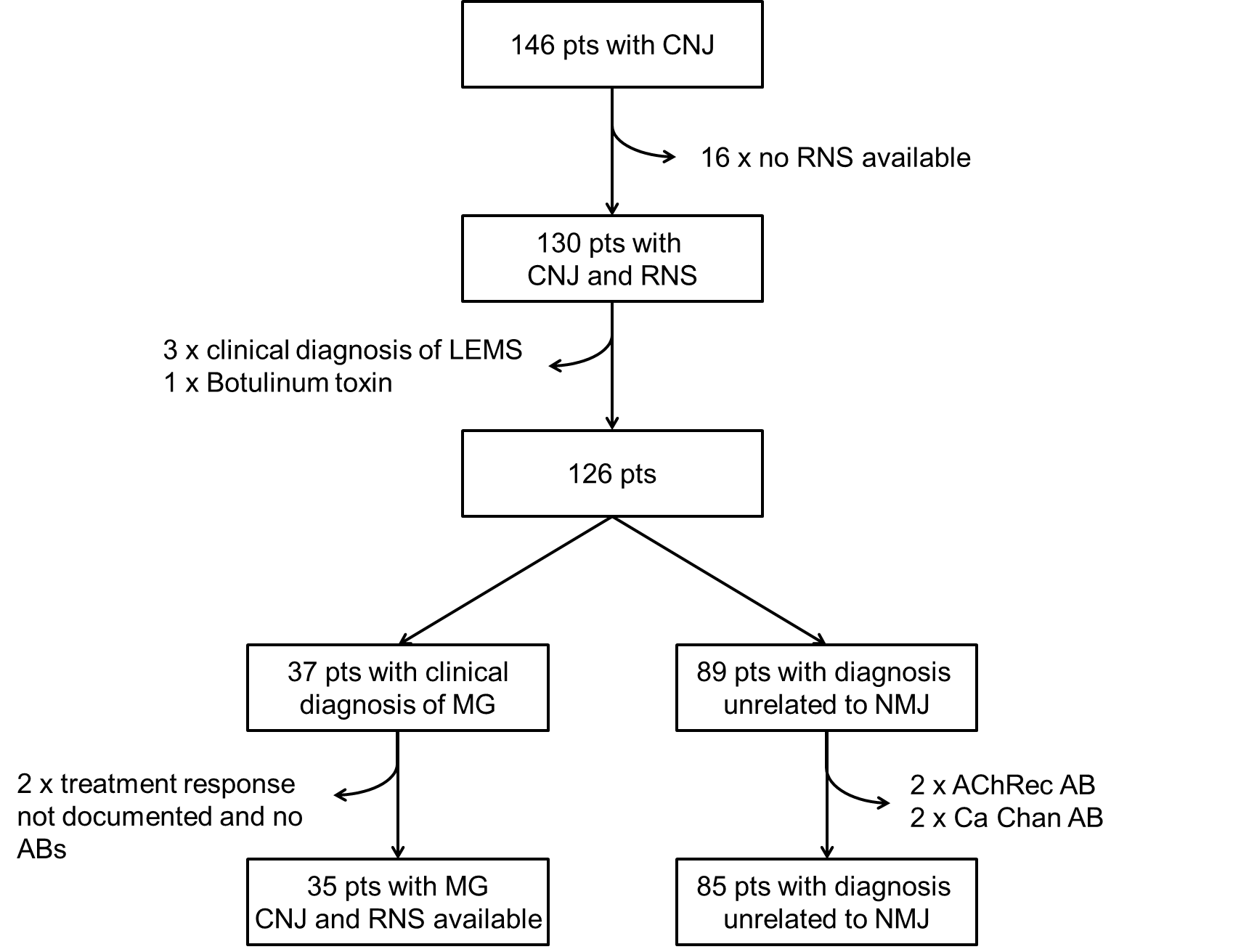


Suppl. Fig. 1: Selection of MG patients and control patients, respectively, from 146 patients in whom CNJ was recorded. ACnRec AB: antibodies to the acetylcholine receptor; Ca Chan AB: antibodies to the PQ type Ca channel.

**Supplementary Table 1**

|  | RNS |  | CNJ |  |
| --- | --- | --- | --- | --- |
|  | + | - | + | - |
| MG | 20 | 15 | 25 | 10 |
| No disorder of NMJ | 9 | 76 | 2 | 83 |

Raw counts for positive (“+”) or negative (“-“) results for the two techniques (RNS and CNJ) in patients with myasthenia gravis and patients without any disorder of the neuromuscular junction.

**Supplementary Table 2**

|  | | CNJ | | total |
| --- | --- | --- | --- | --- |
|  |  | + | - |  |
| RNS | + | 19 | 1 | 20 |
|  | - | 6 | 9 | 15 |
| total | | 25 | 10 | 35 |

Cross table with raw counts for positive (“+”) or negative (“-“) results for the two techniques (RNS and CNJ) in patients with myasthenia gravis (MG).

**Supplementary Table 3**

|  | | CNJ | | total |
| --- | --- | --- | --- | --- |
|  |  | + | - |  |
| RNS | + | 0 | 9 | 9 |
|  | - | 2 | 74 | 76 |
| total | | 2 | 83 | 85 |

Cross table with raw counts for positive (“+”) or negative (“-“) results for the two techniques (RNS and CNJ) in patients without myasthenia gravis (MG).

**Supplementary Table 4**

|  |  | CNJ | | | RNS | | | difference CNJ-RNS | | |
| --- | --- | --- | --- | --- | --- | --- | --- | --- | --- | --- |
|  |  | p | pmin | pmax | p | pmin | pmax | p | pmin | pmax |
| Sens | bootstrap | 0.714 | 0.559 | 0.861 | 0.571 | 0.400 | 0.737 | 0.143 | 0 | 0.289 |
|  | Wilson score |  | 0.535 | 0.848 |  | 0.395 | 0.732 |  |  |  |
| Spec | bootstrap | 0.976 | 0.944 | 1 | 0.894 | 0.864 | 0.970 | 0.082 | -0.01 | 0.122 |
|  | Wilson score |  | 0.910 | 0.996 |  | 0.804 | 0.947 |  |  |  |
| PPV | bootstrap | 0.926 | 0.811 | 1 | 0.690 | 0.565 | 0.897 | 0.236 | 0.006 | 0.377 |
|  | Wilson score |  | 0.742 | 0.987 |  | 0.490 | 0.840 |  |  |  |
| NPV | bootstrap | 0.892 | 0.836 | 0.953 | 0.835 | 0.774 | 0.917 | 0.057 | 0.006 | 0.099 |
|  | Wilson score |  | 0.807 | 0.944 |  | 0.739 | 0.902 |  |  |  |
| Youden | bootstrap | 0.691 | 0.530 | 0.840 | 0.466 | 0.315 | 0.670 | 0.225 | 0.044 | 0.358 |
|  | Shan |  | 0.525 | 0.826 |  | 0.294 | 0.635 |  |  |  |
| PSI | bootstrap | 0.818 | 0.696 | 0.929 | 0.525 | 0.400 | 0.763 | 0.294 | 0.037 | 0.441 |
|  | Shan |  | 0.652 | 0.914 |  | 0.332 | 0.689 |  |  |  |

Confidence intervals for statistical indices as calculated with the bootstrap method and reported in the main manuscript, and as cross-checked with the Wilson Score method with continuity correction and with a method originally developed for the Youden index.
